# Long non-coding RNA SPRY4-IT1 as a promising indicator for three field lymph-node dissection of thoracic esophageal carcinoma

**DOI:** 10.1186/s13019-021-01433-x

**Published:** 2021-03-23

**Authors:** Peng Qie, Qifan Yin, Xuejiao Xun, Yongbin Song, Shaohui Zhou, Huining Liu, Junpeng Feng, Ziqiang Tian

**Affiliations:** 1 Hebei Medical University, Shijiazhuang, 050017 Hebei Province People’s Republic of China; 2 Hebei General Hospital, 348,West He-Ping Road, Shijiazhuang, 050051 Hebei Province People’s Republic of China; 3 Hebei Chest Hospital, Shijiazhuang, 050041 Hebei Province People’s Republic of China; 4 Hebei Medical University Fourth Affiliated Hospital and Hebei Provincial Tumor Hospital, Shijiazhuang, 050035 Hebei Province People’s Republic of China; 5 Thoracic Surgery, The Fourth Hospital of Hebei Medical University, Shijiazhuang, 050035 Hebei Province People’s Republic of China

**Keywords:** Esophageal carcinoma, SPRY4-IT1, Biomarker, Three field lymph-node dissection

## Abstract

**Background:**

Esophageal squamous cell carcinoma(ESCC) is one of the most common tumors worldwide. Esophagectomy with three-field lymph node dissection(3FLND) is the radical surgical procedure for esophageal cancer. However, 3FLND is not widely used due to it’s higher mortality rate and higher incidence of postoperative complications. There is an urgent need to identify novel biomarkers that can guide the most proper lymph-node dissection in esophageal cancer patients.

**Method:**

Ninety-two patients with thoracic ESCC undergoing 3FLND were enrolled into our study from the Department of Thoracic Surgery of the Fourth Hospital affiliated to the Hebei Medical University and Hebei General Hospital between Jun 2011 and Dec 2015. Retrospectively collected data from these 92 patients was used to explore the relationship between the lymph-node metastasis、recurrence and the SPRY4-IT1 expression level and to determine whether 3FLND should be performed in patients with thoracic ESCC.

**Results:**

The findings revealed that the SPRY4-IT1 expression was significantly higher in esophageal cancer tissues than in adjacent noncancerous tissues. (*P* < 0.01). Furthermore, the high expression of SPRY4-IT1 was significantly correlated with tumor differentiation (*P* = 0.029), T classification (*P* = 0.013), lymph node metastasis(*P* = 0.022) and pathological stage (*P* = 0.001). The increased expression of SPRY4-IT1 was associated with a higher risk of cervical and superior mediastinal lymph-node metastasis(*P* = 0.039).However, no significant association was observed between the risk of cervical and superior mediastinal lymph-node recurrence and the SPRY4-IT1 expression level in the thoracic ESCC patients performed 3FLND(*P* = 0.509).

**Conclusions:**

Our data support the assumption that the high expression of SPRY4-IT1 is associated with a high risk of lymph node metastasis and it has potential application as a indicator for guiding on three-field lymph node dissection in patients with thoracic ESCC. Randomized controlled trials with a large cohort of patients will be needed to confirm this conclusion in the future.

## Introduction

Esophageal cancer(EC) is one of the deadliest malignant tumors worldwide. It is the eighth most common cancer and the sixth most common cause of death [1, 2]. Lymph node metastasis is an independent prognostic factor for patients with esophageal cancer [3]. Therefore, esophageal cancer surgery includes removal of the primary lesion and lymph node dissection. Proper lymph node dissection is vital, but the optimal extent of lymph node dissection, namely two field (mediastinal and abdominal stations) versus three field (cervical, mediastinal, and abdominal stations) remains controversial.

The submucosal layer of the esophagus includes abundant crisscrossed lymphatic vessels networks, and early stage esophageal cancer invasion into the submucosa may thus have multiple metastatic LN stations [4, 5]. To prevent postoperative recurrence and lymph-node metastasis and improve long-term survival, extensive radical lymphadenectomy, such as three-field lymph node dissection (3FLND), was introduced for patients with thoracic esophageal carcinoma [6]. Esophagectomy with 3FLND has been proposed for decades, and now being the main surgical procedure for esophageal cancer in Japan. Although many studies have confirmed its survival benefit [7, 8], 3FLND is not widely used in other countries including China because it is associated with a higher mortality rate and a higher incidence of postoperative complications [9]. There is an urgent need to identify novel biomarkers that can help to select patients with a high risk of cervical lymph-node metastasis and esophageal cancer recurrence. Such biomarkers could provide clinical clues for performing proper lymph-node dissection in esophageal squamous cell carcinoma(ESCC).

Recently, numbers of studies have found that long non-coding RNAs (lncRNAs), which refer to a class of RNA transcripts of greater than 200 nucleotides without protein coding function, are playing a role in tumor suppressing or cancer-promoting [10]. With the deepening research of lncRNA function, the study revealed that there are a large number of abnormal expression of lncRNAs in esophageal cancer [11–13], which play an important role in the development and progression of esophageal carcinoma. SPRY4 Intronic transcript 1 (SPRY4-IT1) is a 708-nucleotide-long lncRNA, which comes from the chromosome 5q31.3 [14]. It could influence both cell proliferation and apoptosis. SPRY4-IT1 was previously reported to be up-regulated in melanoma, gastric cancer,breast cancer, esophageal squamous cell carcinoma and colorectal cancer [14–18]. Meanwhile, some studies reported that SPRY4-IT1 was correlated with lymph node metastasis and poor prognosis [13, 19]. Xie et al. reported that the relative expression level of SPRY4-IT1 was associated with T stage, lymph node metastasis, and advanced pathological stage of ESCC patients, SPRY4-IT1 high expression was correlated with lower overall survival rates and could be an independent prognostic factor in patients with ESCC [13]. The objective of this study was to investigate the expression of SPRY4-IT1 in ESCC by qRT-PCR, and to further assess its correlation with lymph node metastasis and poor prognosis and thus guide the most appropriate lymph node dissection strategies for ESCC.

## Materials and methods

### Patients

The study included 92 patients with squamous cell carcinoma of the thoracic esophagus. All patients underwent R0 esophagectomy in the Department of Thoracic Surgery of the Fourth Hospital affiliated to the Hebei Medical University and Hebei General Hospital between Jun 2011 and Dec 2015. Primary cancer tissues and paired adjacent noncancerous tissues(≥2 cm from the edge of carcinoma) were snap-frozen in liquid nitrogen immediately after resection and then stored at − 80 °C for RNA extraction. No patient received chemotherapy、radiotherapy、immunotherapy and traditional Chinese medicine prior to specimen collection. The patients were enrolled into our research according to the following criteria: 1) adult patients with pathologically confirmed esophageal squamous cell carcinoma;2) All of the studied patients underwent radical esophagectomy;3) No treatment strategy was performed before surgery:4) Age of 18 to 75 years. The exclusion criteria were as follows:1) The patients merged with other cancers;2) Cervical esophageal squamous cell carcinoma;3) Patients with severe hypertension、severe pulmonary function injury、massive myocardial infarction、cardiac function ≥ level 2(NYHA)、psychiatric history and severe diabetes complications;4) Patients participated in other clinical trial within the first 4 weeks.

Among the 92 patients with esophageal squamous cell carcinoma,54 patients were male, and 38 patients were female. The range of the patients’ ages at the time of diagnosis was 30–75 years (median, 56 years). The postoperative pathological stages were determined according to the UICC TNM staging system. 35 patients at early stage (TNM stages I), 57 patients at advanced stage (TNM stages II-III);52 patients included lymph-node metastasis,40 patients didn’t have lymph-node metastasis. No residual cancer cells were detected under the upper and lower cutting edge or the lateral margin. The esophageal cancer tissues and paired adjacent noncancerous tissues were confirmed via a pathological examination. This study was approved by the Medical Ethics Committee of the Fourth Hospital affiliated to the Hebei Medical University, and the consent in written form was obtained from all patients.

### Surgical procedure

The scope of operation in three-field lymph node dissection(3FLND) included neck, chest, and abdomen. The specific surgical procedures were as follows:

The chest surgery: 1) The patients were intubated with single-lumen endotracheal tubes and were set in the lateral position. We commonly set a 12 mm trocar at the 9th intercostal space at the posterior axillary line for thoracoscope, a 12 mm trocar at the 7th intercostal space at posterior axillary line, two 5 mm trocars at 4th and 7th intercostal spaces at the anterior axillary line (insufflation of carbon dioxide 6–10 mmHg). 2) Mediastinal pleura was opened along the right vagus nerve and posterior margin of the upper esophagus, identify the root of the right recurrent laryngeal nerve (RLN), expose and protect the right RLN, dissect the right RLN and thoracic paraesophageal lymph nodes (LNs),completely mobilize the upper esophagus. 3) The azygos vein arch was mobilized and resected. 4) We freed the middle and lower segment of the esophagus. The lymph nodes along with the paraesophageal、hilar and esophageal hiatus were dissected. 5) Expose the left RLN, dissect the LNs of the left RLN, dissect the LNs under the carina and completely stop bleeding. The esophagus was dissected from the diaphragm to the apex of the chest, along with the mediastinal and hilar lymph nodes dissection, including the bilateral RLNs and subcarinal LNs.

The abdominal and cervical surgery: 1) Place the patients in the supine position and separate legs, expose the small bend in the stomach; cut off the left gastric artery, and lymph nodes along the left gastric artery、hepatic artery and the celiac axis were dissected. 2) The stomach was mobilized with reserve of the right gastroepiploic artery, meanwhile, dissect the perigastric LNs, separate the esophageal hiatus and mobilize the abdominal esophagus. 3) Make an 5 cm incision in the anterior border of the sternocleidomastoid muscle of the left cervix, separate the sternocleidomastoid, dissect the LNs in the left lower cervical esophagus [included left cervical paraesophageal (101) and supraclavicular (104)] and cut off the cervical esophagus at the level of thoracic inlet. 4) Make a 5 cm subxiphoid vertical incision in the centre of the abdomen, remove the stomach and esophagus with lesion, a tubular stomach was reconstructed using linear cutting stapler, lift the tubular stomach to the left neck by way of the esophageal hiatus and esophageal bed, a circular stapler was inserted to anastomose the greater curvature side of the tubular stomach and the cervical esophageal. Then, a linear cutting stapler was used to close the proximal end of the tubular stomach. 5) Make an 5 cm incision in the anterior border of the sternocleidomastoid muscle of the right cervix and dissect the LNs in the right lower cervical esophagus [included right cervical paraesophageal (101) and supraclavicular (104)].

### RNA extraction and qRT-PCR

Total RNA was extracted from specimens of esophageal cancer tissues and paired adjacent noncancerous tissues using Trizol reagent (Invitrogen,Carlsbad, CA).RNA was reversely transcribed into cDNAs using the SuperScript first-strand synthesis system (Invitrogen, Carlsbad, CA, USA). To evaluate SPRY4-IT1 expression levels, we used quantitative real time polymerase chain reaction (qRT-PCR) with 2 × TaqMan Premix Ex Taq (Takara, Japan), using an ABI7900 system (Applied Biosystems, CA,USA). Dissociation curve analysis was used to evaluate the PCR products. Glyceraldehyde 3-phosphate dehydrogenase (GAPDH) was used as an internal control. The comparative 2-ΔΔCt method was used for relative quantification and statistical analysis. The primers of GAPDH and SPRY4-IT1 were synthesized by Sangon Biotech (Shanghai, China). SPRY4-IT1 expression levels were determined using 5′-AGCCACATAAATTCAGCAGA-3′ as a forward primer and 5′-CGATGTAGTAGGATTCCTTTCA3′ as a reverse primer sequence. Results were normalized to GAPDH using forward, 5′-GTCAACGGATTTGGTCTGTATT-3′and reverse 5′-AGTCTTCTGGGTGGCAGTGAT-3′primers.

### Postoperative follow-up and the diagnosis of lymph-node recurrence

All patients underwent follow-up examinations every 3–6 months after surgery. These examinations included Doppler ultrasound, chest and abdominal enhanced CT examination, and, if necessary, PET-CT and endoscopy. Recurrence was defined as apparent recurrence on imaging studies during follow-up. The diagnosis of cervical lymph-node recurrence was mainly made based on the results of a physical examination, Doppler ultrasound, and fine-needle aspiration cytology. The diagnosis of mediastinal lymph-node recurrence was mainly based on CT findings. The diagnosis of abdominal lymph-node recurrence was mainly based on Doppler ultrasound and CT findings.

### Statistical analysis

All statistical analyses were performed using SPSS 21.0 software (SPSS Inc., Chicago, IL, USA). The X^2^ test was used to analyze the relationship between SPRY4-IT1 expression and clinicopathological characteristics. The correlation between lymph-node metastasis、recurrence and SPRY4-IT1 expression was analyzed by X^2^ test or nonparametric test (Mann–Whitney U test). The Kaplan-Meier method was used to calculate the survival curves. Univariate analysis and multivariate analysis were performed using the Cox proportional hazard regression model. A *p* value less than 0.05 was considered statistically significant. All of the experiments were repeated three times.

## Results

### SPRY4-IT1 is highly expressed in esophageal cancer tissues

**Fig. 1:**
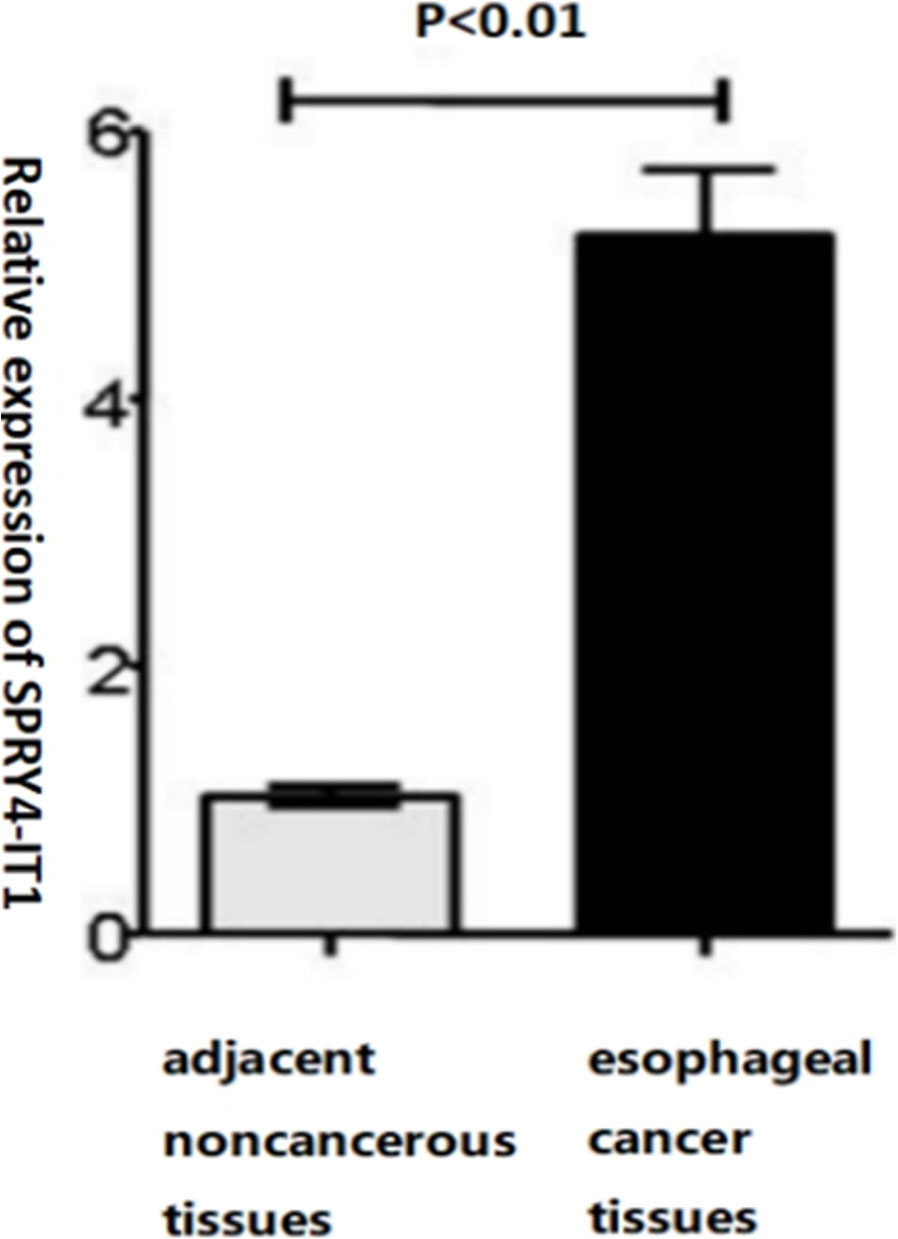
The up-regulated level of SPRY4-IT1 was measured in esophageal cancer tissues compared with adjacent noncancerous

Quantitative RT-PCR was used to measure SPRY4-IT1 expression levels in 92 patients of esophageal cancer tissues and paired adjacent noncancerous tissues. Expression values were normalized to 1 in adjacent noncancerous tissues. As shown in Fig. 1, the results showed that the relative expression of SPRY4-IT1 was significantly higher in esophageal cancer tissues than in adjacent noncancerous tissues. (*P* < 0.01,Fig. 1).

### The relationship between SPRY4-IT1 expression and the clinicopathologic features of esophageal cancer

Then, the 92 patients with ESCC were divided into high and low expression groups by the mean expression level of SPRY4-IT1. The high SPRY4-IT1 expression group (*n* = 47) had SPRY4-IT1 expression levels > mean value and the low SPRY4-IT1 expression group (*n* = 45) had SPRY4-IT1 expression levels < mean value. As shown in Table 1, the high expression of SPRY4-IT1 was correlated with tumor differentiation (*P* = 0.029), T classification (*P* = 0.013), lymph node metastasis(*P* = 0.022) and pathological stage (*P* = 0.001) but not correlated with patient’s age, gender, smoking status, alcohol consumption, tumor location, and lymph-node recurrence(*P* > 0.05). Taken together, these observations suggest that increased SPRY4-IT1 expression is associated with the malignant degree、progression、later stage of ESCC.

**Table 1:** Relationship between SPRY4-IT1 expression and clinicopathological features in patients with ESCC

Features(N)	SPRY4-IT1 expression level		*P* value
	High(n = 47)	Low(n = 45)	
Age group			*P* = 0.686
≤ 60	26	23	
>60	21	22	
Gender			*P* = 0.549
Male	29	25	
Female	18	20	
Smoking status			*P* = 0.537
Ever and current	26	22	
Never	21	23	
Alcohol consumption			*P* = 0.525
Ever and current	24	20	
Never	23	25	
Tumor location			*P* = 0.808
Upper	9	7	
Middle	22	24	
Lower	16	14	
Differentiation			*P* = 0.029
Well	7	16	
Moderate	21	20	
Poor	19	9	
T classification			*P* = 0.013
Tis-2	16	27	
T3–4	31	18	
Lymph node metastasis			*P* = 0.022
Yes	32	20	
No	15	25	
Pathological stage			*P* = 0.001
Stage I	10	25	
Stage II-III	37	20	
Lymph node recurrence			*P* = 0.077
Yes	38	29	
No	9	16	

### Association between the SPRY4-IT1 expression level and lymph-node metastasis

All of the enrolled patients(n = 92) underwent R0 esophagectomy with 3FLND including the cervical, mediastinal, and abdominal lymph nodes. Among 52 patients with lymph-node metastasis, 25 patients harbored cervical and superior mediastinal lymph-node metastasis, the other 27 patients only had others’ site lymph-node metastasis. Among 32 patients with high expression of SPRY4-IT1 harboring lymph-node metastasis, 19 patients (59.4%) had cervical and superior mediastinal lymph-node metastasis and 13 patients (40.6%) only had others’ site lymph-node metastasis that didn’t include cervical and superior mediastinum. Among 20 patients with low expression of SPRY4-IT1 existing lymph-node metastasis,6 patients (30%) had cervical and superior mediastinal lymph-node metastasis and 14 patients (70%) only had others’ site lymph-node metastasis excluded cervical and superior mediastinum. We performed a Chi-squared test to analyze the association between cervical and superior mediastinal lymph-node metastasis and the SPRY4-IT1 expression level. We found that the increased expression level of SPRY4-IT1 was associated with a higher risk of cervical and superior mediastinal lymph-node metastasis(*P* = 0.039, Table 2).

**Table 2:** Association between the SPRY4-IT1 expression level and site of lymph-node metastasis

Site of lymph-node metastasis	SPRY4-IT1 expression level		*P*
	High	Low	
Cervical and superior mediastinal lymph-node	19	6	0.039
Others	13	14	

### Association between the SPRY4-IT1 expression level and lymph-node recurrence

All patients underwent follow-up examinations every 3–6 months after surgery until the end of the postoperative 5-year follow up period or death. A total of 67 patients developed lymph-node recurrence during follow-up. Among 47 patients with the high expression of SPRY4-IT1, 38 patients (80.9%) had lymph-node recurrence. 24 patients (51.1%) had cervical and superior mediastinal lymph node recurrence and 14 patients (27.8%) had others’ site lymph node recurrence excluding the cervical and superior mediastinal areas. Among 45 patients with the low expression of SPRY4-IT1, 29 patients (64.4%) had lymph-node recurrence. 16 patients (35.5%) had cervical and superior mediastinal lymph node recurrence and 13 patients (28.9%) had others’ site lymph node recurrence that didn’t include cervical and superior mediastinal lymph node. We performed a Chi-squared test to analyze the association between the SPRY4-IT1 expression level and cervical and superior mediastinal lymph-node recurrence. The association was not statistically significant in the ESCC patients underwent R0 esophagectomy with 3FLND(*P* = 0.509,Table 3). Therefore, in the thoracic ESCC patients performed 3FLND, no significant association was observed between the risk of cervical and superior mediastinal lymph-node recurrence and the SPRY4-IT1 expression level.

**Table 3:** Association between the SPRY4-IT1 expression level and site of lymph-node recurrence

Site of lymph-node recurrence	SPRY4-IT1 expression level		*P*
	High	Low	
Cervical and superior mediastinal lymph-node	24	16	0.509
Others	14	13	

### The survival analyses about PFS and OS

**Fig. 2:**
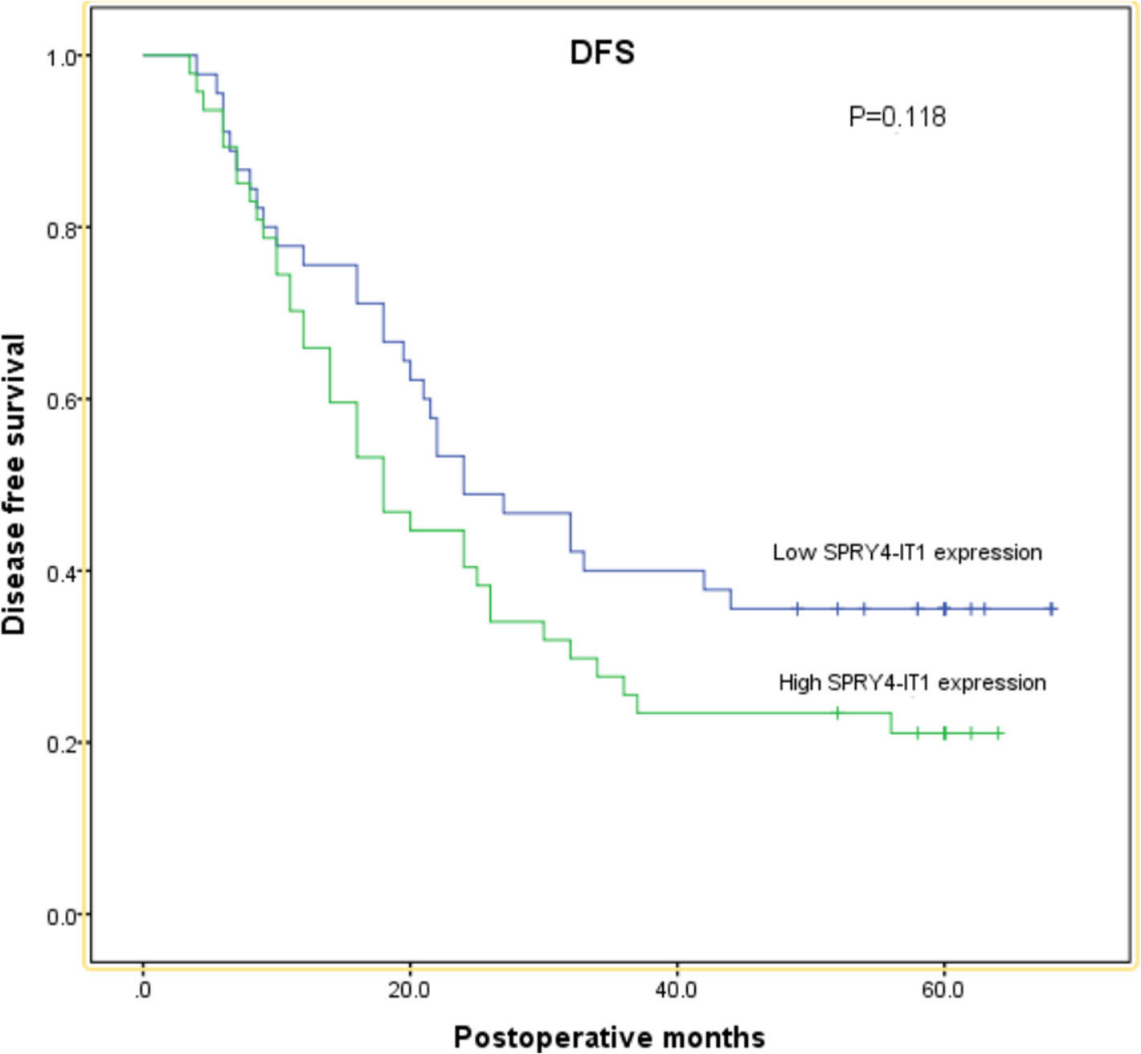
Kaplan–Meier curves for PFS according to the expression of SPRY4-IT1

**Fig. 3:**
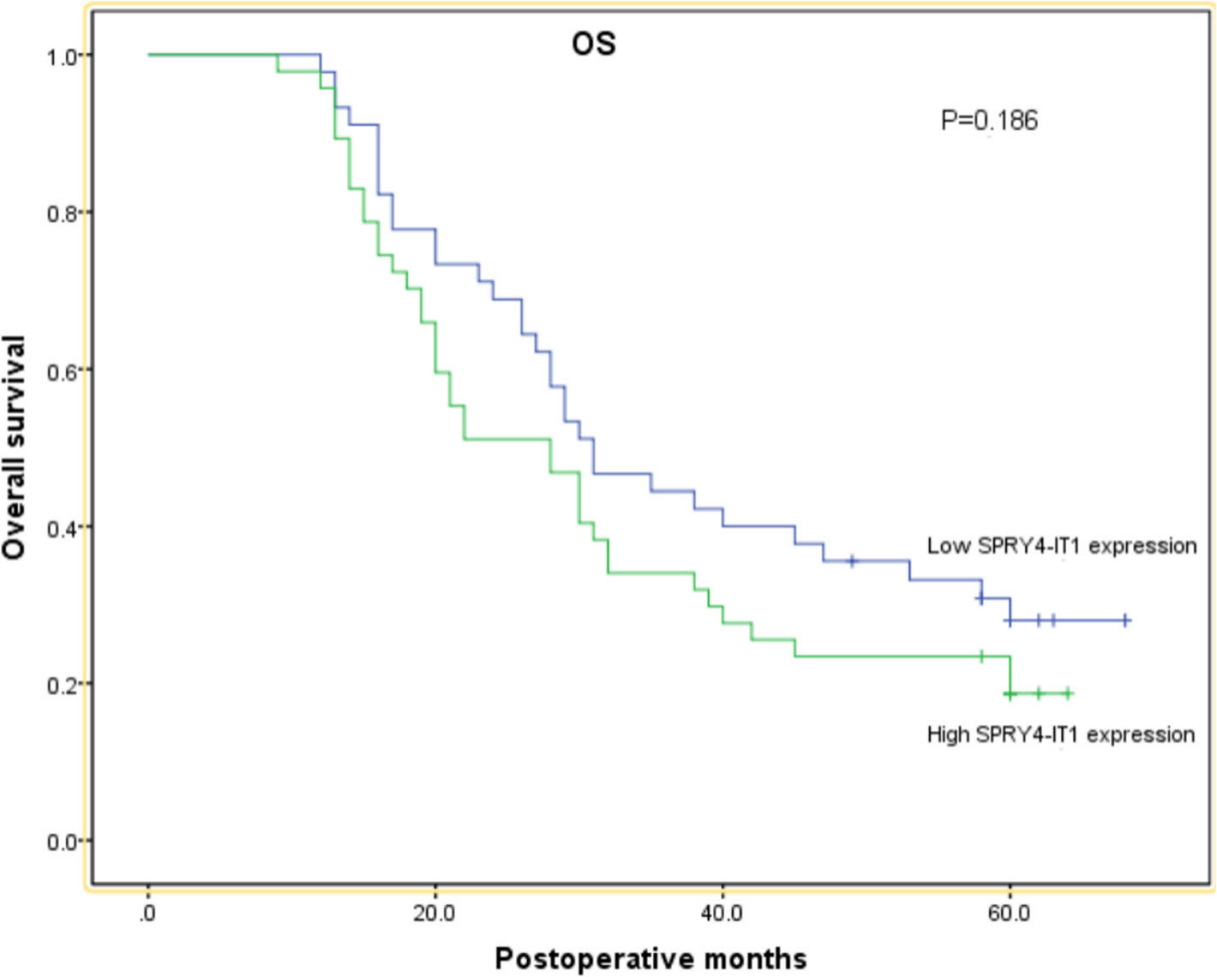
Kaplan–Meier curves for OS according to the expression of SPRY4-IT1

**Of the 92 patients, 55 (60%) patients died within 5 years after surgery, and recurrent disease developed in 67 (72.8%) patients during the follow-up period. The Kaplan–Meier analysis was used to calculate the survival analysis about progression-free survival (PFS) and overall survival (OS). The patients with high SPRY4-IT1 expression had a trend of shorter PFS and OS than patients with low SPRY4-IT1 expression in the ESCC patients underwent 3FLND from the survival curves, but no significant differences were found about PFS and OS(*P*** **= 0.118 and** ***P*** **= 0.186, respectively;Fig.** **2****,Fig.** **3****).**

## Discussion

Lymphatic metastasis is an important metastatic pathway in patients with ESCC. The esophageal submucosa includes abundant LNs and longitudinal lymphatic networks [4],Cancer cells can be transferred along with the complex LN system. In addition, the incidence of metastasis to the extramediastinal nodes in the absence of mediastinal metastasis (jumping metastasis) is high. Moreover, this high rate of jumping metastasis is considered to be a characteristic of lymph-node metastasis in thoracic esophageal cancer [20]. Thus, the enlargement of the area of lymph-node resection seems to be important for the long-term survival of ESCC patients [7]. Patients with esophageal cancer require 3FLND, which helps to improve the accuracy of staging, guiding subsequent treatment, reducing postoperative tumor recurrence, and improving the survival rate of patients [21]. However, compared with two-field LN dissection, three-field LN dissection is a more complicated surgical procedure that requires longer time and exposes the patient to greater trauma. The high rate of postoperative complications, including recurrent laryngeal nerve injury, anastomotic leakage, chylothorax and pulmonary infection, cannot be completely avoided [6, 22, 23]. Therefore, there is an urgent need to identify novel biomarkers that can help to select patients with a high risk of cervical and superior mediastinal lymph-node metastasis and esophageal cancer recurrence. Such biomarkers could provide clinical clues for performing proper lymph-node dissection in ESCC patients.

Previous studies showed that the rate of cervical LN metastasis in patients with esophageal cancer was 16–43% and the recurrence rate is 6–16% [24, 25]. The ability to predict and detect cervical LN metastasis is thus key to improving surgical efficacy in patients with ESCC. In recent years, a increasing number of papers have reported that the expression level of lncRNAs can be used as robust and crucial biomarkers for cancer risk, diagnosis, and prognosis [13, 19]. Xie et al. reported that the high expression level of SPRY4-IT1 was associated with T stage, lymph node metastasis, and advanced pathological stage of ESCC patients [13]. Based on previous researches, our attention then focused on SPRY4-IT1.

In our present study, we determined the expression levels of SPRY4-IT1 in ESCC tissues and matched adjacent noncancerous tissues and observed a significantly higher expression of SPRY4-IT1 in the esophageal carcinoma tissues than in the paired adjacent normal esophageal tissues. We further discovered the high expression level of SPRY4-IT1 was significantly associated with tumor differentiation, T classification, lymph node metastasis and pathological TNM stage, suggesting SPRY4-IT1 contributed to progression and poor prognosis of ESCC. Similar results were also found in other types of cancer, and a higher expression of SPRY4-IT1 predicted poor prognosis in many cancers, such as gastric cancer, colorectal cancer, non-small cell lung cancer, and breast cancer [15, 16, 18, 26]. On the basis of the previous studies, we hypothesized that the high SPRY4-IT1 expression of ESCC patients might be used as a biomarker for guiding on three-field LN dissection.

All of patients(*n* = 92) underwent R0 esophagectomy with 3FLND including the cervical, mediastinal, and abdominal lymph nodes were enrolled into our study. We found that the ESCC patients with the high expression of SPRY4-IT1 showed a higher rate of cervical and superior mediastinal lymph-node metastasis in comparison to the low expression of SPRY4-IT1 group (*P* = 0.039, Table 2).However, no significant association was observed between the cervical and superior mediastinal lymph-node recurrence and the SPRY4-IT1 expression level(*P* = 0.509,Table 3). Even, there was no obvious difference between the lymph-node recurrence and the SPRY4-IT1 expression statue in the ESCC patients with 3FLND esophagectomy(*P* = 0.077,Table 1). This reflected that the high expression of SPRY4-IT1 is associated with a higher risk of cervical and superior mediastinal lymph-node metastasis and that this could be mitigated by radical three-field lymph node dissection surgery. **From the survival analysis, the patients with high SPRY4-IT1 expression had a trend of shorter PFS and OS than patients with low SPRY4-IT1 expression,which indicated that the high expression of SPRY4-IT1 might have a unfavourable effect on the ESCC patients’ prognosis. But no significant differences were observed in the ESCC patients with 3FLND.Thus, patients with high SPRY4-IT1 expression might benefit from 3FLND esophagectomy.** Based on our work, the high expression of SPRY4-IT1 might be used as a promising indicator for selecting three-field lymph node dissection for thoracic ESCC patients.

Some limitations of the present study should be addressed. First, this was a retrospective investigation with a relatively small population. In the following study, a large scale、randomized controlled trial will be needed to validate our conclusion. Second, only 3FLND ESCC patients were included. It is also important to investigate the association between lymph-node recurrence and the high expression of SPRY4-IT1 in two-field lymph node dissection group.

## Conclusion

In conclusion, Our data support the assumption that the high expression of SPRY4-IT1 is associated with a high risk of lymph node metastasis and it has potential application as a indicator for guiding on three-field lymph node dissection in patients with thoracic ESCC. Randomized controlled trials with a large cohort of patients will be needed to confirm this conclusion in the future.

## Data Availability

The datasets used and analysed during the current study are available from the corresponding author on reasonable request.

## Abbreviations

EC: esophageal cancer
ESCC: esophageal squamous cell carcinoma
LN: lymph node
3FLND: three-field lymph node dissection
lncRNAs: long non-coding RNAs
SPRY4-IT1: SPRY4 Intronic transcript 1
qRT-PCR: quantitative real time polymerase chain reaction
CT: computed tomography
PFS: progression-free survival
OS: Overall survival
